# Synthetic genistein derivatives as modulators of glycosaminoglycan storage

**DOI:** 10.1186/1479-5876-10-153

**Published:** 2012-07-30

**Authors:** Anna Kloska, Magdalena Narajczyk, Joanna Jakóbkiewicz-Banecka, Grzegorz Grynkiewicz, Wiesław Szeja, Magdalena Gabig-Cimińska, Grzegorz Węgrzyn

**Affiliations:** 1 Department of Molecular Biology, University of Gdańsk, Kładki 24, Gdańsk, 80-822, Poland; 2 Laboratory of Electron Microscopy, University of Gdańsk, Kładki 24, Gdańsk, 80-822, Poland; 3 Pharmaceutical Research Institute, Rydygiera 8, Warsaw, 01-793, Poland; 4 Department of Chemistry, Silesian Technical University, Gliwice, 44-100, Poland; 5 Laboratory of Molecular Biology, (affiliated with University of Gdańsk), Institute of Biochemistry and Biophysics, Polish Academy of Sciences, Kładki 24, Gdańsk, 80-822, Poland

**Keywords:** Mucopolysaccharidoses, Substrate reduction therapy, Synthetic derivatives of genistein

## Abstract

**Background:**

Mucopolysaccharidoses (MPS) are severe metabolic disorders caused by accumulation of undegraded glycosaminoglycans (GAGs) in lysosomes due to defects in certain lysosomal hydrolases. Substrate reduction therapy (SRT) has been proposed as one of potential treatment procedures of MPS. Importantly, small molecules used in such a therapy might potentially cross the blood–brain barrier (BBB) and improve neurological status of patients, as reported for a natural isoflavone, 5, 7-dihydroxy-3- (4-hydroxyphenyl)-4 *H*-1-benzopyran-4-one, also known as genistein. Although genistein is able to cross BBB to some extent, its delivery to the central nervous system is still relatively poor (below 10% efficiency). Thus, we aimed to develop a set of synthetically modified genistein molecules and characterize physicochemical as well as biological properties of these compounds.

**Methods:**

Following parameters were determined for the tested synthetic derivatives of genistein: cytotoxicity, effects on cell proliferation, kinetics of GAG synthesis, effects on epidermal growth factor (EGF) receptor’s tyrosine kinase activity, effects on lysosomal storage, potential ability to cross BBB.

**Results:**

We observed that some synthetic derivatives inhibited GAG synthesis similarly to, or more efficiently than, genistein and were able to reduce lysosomal storage in MPS III fibroblasts. The tested compounds were generally of low cytotoxicity and had minor effects on cell proliferation. Moreover, synthetic derivatives of genistein revealed higher lipophilicity (assessed *in silico*) than the natural isoflavone.

**Conclusion:**

Some compounds tested in this study might be promising candidates for further studies on therapeutic agents in MPS types with neurological symptoms.

## Background

Mucopolysaccharidoses (MPS) are rare lysosomal storage disorders caused by deficiencies in activities of several different lysosomal hydrolases. Mutations in genes coding for these enzymes lead to defects in degradation of glycosaminoglycans (GAGs) [1,2]. Excessive accumulation of undegraded GAGs in lysosomes causes severe problems in most tissues and organs and usually leads to death in childhood [2]. Currently, only two therapeutic procedures are available for treatment of some of MPS types: bone marrow (or hematopoietic cell) transplantation and enzyme replacement therapy (ERT) [3]. The former procedure is the therapy of choice in MPS I, as it can halt neurocognitive decline when performed early, preferably before the age of 2.5 years [4]. However, efficacy has only been demonstrated in MPS I and MPS VI, and not in MPS III [3,4]. The latter treatment is based on administration of the lacking enzyme, and it is currently available for MPS I, MPS II and MPS VI [5]. Although this treatment is to some extent effective in management of somatic symptoms of the disease, in many MPS types (MPS IH, MPS II, all MPS III subtypes, MPS VII) central nervous system (CNS) is also affected, and ERT seems to be of low efficacy in treatment of neurological symptoms because of the poor delivery of enzyme molecules to CNS across the blood–brain barrier (BBB) [4,6].

Substrate reduction therapy (SRT) is one of putative alternative methods for MPS treatment [6]. A specific kind of SRT for MPS, based on administration of genistein (5, 7-dihydroxy-3- (4-hydroxyphenyl)-4 *H*-1-benzopyran-4-one also known as 4', 5, 7-trihydroxyisoflavone), has been proposed [7]. *In vitro*, genistein significantly inhibits GAG synthesis and results in a decrease in lysosomal storage in MPS cells [7]. Expression of genes coding for enzymes involved in GAG synthesis might be controlled by signalling pathways dependent on a tyrosine kinase activity of epidermal growth factor receptor (EGFR) [8,9], and genistein has been reported to inhibit this enzymatic activity [10]. In fact, genistein-mediated SRT was reported to act due to inhibition of phosphorylation of EGFR [11] and subsequent putative modulation of gene expression. Therefore, this specific kind of SRT has been named ‘gene expression-targeted isoflavone therapy’ or GET IT [12-14].

Since genistein was reported to cross the BBB to some extent [15], it has been suggested that GET IT might be effective in treatment of neurological symptoms of MPS. In fact, *in vivo* studies performed with MPS IIIB mouse model revealed a significant reduction of lysosomal storage in liver of MPS IIIB mice treated with genistein for 8 weeks [16], and correction of the abnormal behavior in a long-term (9 month) experiment with high genistein dose (160 mg/kg/day) [17]. Additionally, in both open label and placebo-controlled studies with MPS III patients treated with a genistein-rich soy extract at relatively low doses (5–15 mg/kg/day), some – though only limited – positive effects on urinary and plasma GAG levels, hair morphology, cognitive functions and behavior were reported [18-22]. This low efficacy of GET IT in clinical trials, which is in contrast to promising results of experiments performed *in vitro* and with mice, has recently been suggested to be due to low genistein doses in former studies (5–15 mg/kg/day in clinical studies vs. 160 mg/kg/day in animal-based experiments) [14]. Nevertheless, other mechanisms, like limited effects of genistein in human body and/or low efficiency of crossing BBB by this isoflavone (this efficiency was estimated to be below 10% in rats [15]), could not be excluded.

Simultaneously to clinical trials, further laboratory experiments on GET IT have been performed and it was demonstrated that some other natural isoflavones, or even flavonoids, may also cause an inhibition of GAG synthesis and reduction of their accumulation in MPS cells [23,24]. Therefore, one might speculate that chemical modification(s) of genistein might improve either its efficiency in GAG synthesis inhibition or efficiency in crossing BBB. If so, GET IT could be of higher efficacy in MPS patients. In this study, we aimed to test a series of synthetic derivatives of genistein in terms of efficiency of GAG synthesis inhibition and potential ability to cross BBB.

## Methods

### Chemicals

Genistein was obtained at the Pharmaceutical Research Institute (Warsaw, Poland) on the pilot plant scale, according to proprietory method [25]. A method for regioselective derivatization of its phenolic groups was designed, based on unique, stable tetrabutylammonium salt [26]. Preparations of its synthetic derivatives were already described in connection with study of antiproliferative activity [27]. The derivatives listed in Table 1 have also been claimed as modulators of GAG storage in CNS (United States Patent no. US 8,178,609 B2; date of patent: May 15, 2012; inventors: Grynkiewicz G., Wegrzyn G., Szechner B., Tylki-Szymanska A., Wegrzyn A., Jakobkiewicz-Banecka J., Baranska S., Czartoryska B., Piotrowska E., title: Isoflavones for treating mucopolysaccharidoses). Stock solutions were prepared in dimethylformamide (DMF). MTT reagent (3-(4,5-dimethylthiazol-2-yl)-2,5- diphenyltetrazolium bromide), purchased from Sigma (Germany), was dissolved in RPMI-1640 medium without phenol red (Sigma, Germany). Phosphate Bufered Saline (PBS), dimethylsulfoxide (DMSO) and dimethylformamide (DMF) were from Sigma (Germany).

**Table 1:** Synthetic derivatives of genistein

**Symbol**	**IUPAC systematic name**	**Molecular formula**	**Molecular weight**
IFG-001	4-(5,7-dihydroxy-4-oxo-4 H-chromen-3-yl)phenyl 2-aminobenzoate	C_22_H_15_NO_6_	389.367
IFG-018	5-hydroxy-3-(4-hydroxyphenyl)-4-oxo-4 H-chromen-7-yl heptadecanoate	C_31_H_40_O_6_	508.65
IFG-021	7-O-[(2,3,4,6-tetra-O-acetyl-β-D-galactopyranosyl)-1,4-(6-O-acetyl-hex-2-ene-α-D- erythropyranosyl)]-5-hydroxy-3-(4-hydroxyphenyl)-4-H-chromen-4-one	C_37_H_38_O_18_	770.689
IFG-027	5-hydroxy-3-(4-hydroxyphenyl)-7-(prop-2-en-1- yloxy)-4 H-chromen-4-one	C_18_H_14_O_5_	310.30
IFG-032	4-[5,7-bis(acetyloxy)-4-oxo-4 H-chromen-3-yl]phenyl acetate	C_21_H_16_O_8_	396.35
IFG-034	5-hydroxy-3-(4-hydroxyphenyl)-4-oxo-4 H-chromen-7-yl)-2-acetyloxybenzoate	C_24_H_16_O_8_	432.39
IFG-035	5,7-bis(prop-2-en-1-yloxy)-3-[4-(prop-2-en-1- yloxy)phenyl]-4 H-chromen-4-one	C_22_H_22_O_5_	390.43
IFG-036	ethyl 2-((5-hydroxy-3-(4-hydroxyphenyl)-4-oxo-4 H-chromen-7-yl)oxy)acetate	C_19_H_16_O_7_	356.33
IFG-037	tert-butyl-2-((5-hydroxy-3-(4-hydroxyphenyl)-4-oxo-4 H-chromen-7-yl)oxy)acetate	C_21_H_20_O_7_	384.382
IFG-038	tetrabutylamonium 5-{[5-hydroxy-3-(4-hydroxyphenyl)-4-oxo-4 H-chromen-7-yl]oxy}-5-oxopentanoate	C_36_H_51_NO_8_	625.1
IFG-042	tert-butyl 2-[(3-{4-[2-(tert-butoxy)-2- oxoethoxy]phenyl}-5-hydroxy-4-oxo-4 H-chromen-7-yl)oxy]acetate	C_27_H_30_O_9_	498.52
IFG-043	tert-butyl 2-[(3-{4-[2-(tert-butoxy)-2-oxoethoxy]phenyl}-5-hydroxy-4-oxo-4 H-chromen-7-yl)oxy]acetate	C_22_H_16_O_5_	360.36
IFG-046	2(2(2-((4-oxo-4 H-chromen-7-yl)oxy)ethoxy)ethoxy)ethyl)-4-methylbenzenesulfonate	C_28_H_28_O_10_S	556.14
IFG-048	tert-butyl 2-[4-(5,7-dihydroxy-4-oxo-4 H-chromen-3-yl)phenoxy]acetate	C_21_H_20_O_7_	384.382
IFG-050	5-hydroxy-3-(4-hydroxyphenyl)- 7-O-(epoxymethyl)- 4-H-chromen-4-one	C_18_H_14_O_6_	326.30
IFG-051	7-(benzyloxy)-5-hydroxy-3-[4-(prop-2-en-1- yloxy)phenyl]-4 H-chromen-4-one	C_25_H_20_O_5_	400.42
IFG-052	4-[5-hydroxy-4-oxo-7-(prop-2-en-1-yloxy)-4 H- chromen-3-yl]phenyl 2-(acetyloxy)benzoate	C_27_H_20_O_8_	472.44
IFG-053	2-(3-{4-[7-(benzyloxy)-5-hydroxy-4-oxo-4 H-chromen-3-yl]phenoxymethyl}-5-(1-cyano-1-methylethyl)phenyl)-2-methylpropanenitrile	C_37_H_32_N_2_O_5_	584.66
IFG-054	2-{[5-hydroxy-3-(4-hydroxyphenyl)-4-oxo-4 H-chromen-7-yl]oxy}acetic acid	C_17_H_12_O_7_	328.27
IFG-060	5-{4-[7-(benzyloxy)-5-hydroxy-4-oxo-4 H-chromen-3-yl]phenoxy}-5-oxopentanoic acid	C_27_H_22_O_8_	474.47
IFG-061	4-[7-(benzyloxy)-5-hydroxy-4-oxo-4 H-chromen-3-yl]phenyl 1-sodium pentanedioate	C_27_H_21_O_8_Na	480.45
IFG-062	5-hydroxy-3-(4-hydroxyphenyl)-7-[(4- methoxyphenyl)methoxy]-4 H-chromen-4-one	C_23_H_18_O_6_	390.39
IFG-063	[5-hydroxy-3-(4-hydroxyphenyl)-4-oxo-4 H-chromen-7-yl] prop-2-en-1-yl carbonate	C_19_H_14_O_7_	354.31
IFG-064	7-(benzyloxy)-5-hydroxy-3-[4-(propan-2- yloxy)phenyl]-4 H-chromen-4-one	C_25_H_22_O_5_	402.44
IFG-065	7-(benzyloxy)-5-(propan-2-yloxy)-3-[4-(propan-2-yloxy)phenyl]-4 H-chromen-4-one	C_28_H_28_O_5_	444.52
IFG-066	5,7-dihydroxy-3-[4-(propan-2-yloxy)phenyl]-4 H- chromen-4-one	C_18_H_16_O_5_	312.32
IFG-067	4-[7-(benzyloxy)-5-hydroxy-4-oxo-4 H-chromen-3-yl]phenyl acetate	C_24_H_18_O_6_	402.4
IFG-070	methyl 2-{[5-hydroxy-3-(4-hydroxyphenyl)-4-oxo-4 H-chromen-7-yl]oxy}acetate	C_18_H_14_O_7_	342.16
IFG-071	5-{[5-hydroxy-3-(4-hydroxyphenyl)-4-oxo-4 H-chromen-7-yl]oxy}pentyl acetate	C_22_H_22_O_7_	398.40
IFG-072	5-hydroxy-3-(4-hydroxyphenyl)-7-(3-hydroxypropoxy)-4 H-chromen-4-one	C_18_H_16_O_6_	328.31
IFG-073	5-hydroxy-7-(2-hydroxyethoxy)-3-(4-hydroxyphenyl)-4 H-chromen-4-one	C_17_H_14_O_6_	314.22
IFG-074	tetrabutylamonium 2-{[5-hydroxy-3-(4-hydroxyphenyl)-4-oxo-4 H-chromen-7-yl]oxy}acetate	C_33_H_47_O_7_N	569.73

### Cell lines and culture conditions

Fibroblast cell lines obtained from MPS IIIA and MPS IIIB patients were used in all experiments. Human Dermal Fibroblast adult line (HDFa; Cascade Biologics, Portland, OR, USA) was used as a healthy control line. Cells were grown in Dulbecco's Modified Eagle Medium (DMEM) supplemented with 10% Fetal Bovine Serum (FBS) and 1 x Antibiotic and Antimycotic Solution (all purchased from Sigma, Germany) at 37 °C in humidified 5% CO_2_ atmosphere. GAG synthesis experiments were performed using Minimal Essential Medium without inorganic sulfates (MEM, Joklik’s modified; Sigma, Germany).

### Cytotoxicity and proliferation assay

Cytotoxicity and cell proliferation was assessed using MTT assay. Cells were seed in 96-well plates in a number of 6 x 10^3^ cells per well (cytotoxicity assay) or 10^3^ cells per well (proliferation assay). After an overnight incubation, growth medium was substituted with medium supplemented with appropriate concentrations of genistein synthetic derivatives or 0.05% DMF as a control and cells were incubated for 24- or 48-hours (cytotoxicity assay) or for 7-days (proliferation assay). Then, medium was substituted with MTT solution (1 mg/ml in RPMI-1640 medium) and following 2-hour incubation at 37°C the amount of a purple formazan product dissolved in DMSO was quantified by measuring the absorbance at 550 nm. LC_50_ (cytotoxicity assay) or IC_50_ (proliferation assay) index values were determined relative to nontreated cultures (incubated with DMF only).

### Measurement of kinetics of GAG synthesis

Cells seed in a number of 2 x 10^4^ cells per well (48-well plate) were incubated in growth medium overnight. Then, the medium was substituted with another one, containing appropriate concentrations of genistein synthetic derivatives or 0.05% DMF as a control, and cells were grown for 48 hours. In the next step, the medium was substituted with growth medium without inorganic sulfates (MEM, Joklik’s modified) mixed with standard DMEM medium (1:1) supplemented with FBS. GAGs were labeled with 20 μCi/ml of H_2_[^35^ S]O_4_ (Hartmann Analytic) for 24 hours. Cells washed with PBS were digested for 3 hours with 0.03% papain (prepared in 100 mM sodium acetate with 5 mM L-cysteine, pH 7.0) (Merck KGaA, Darmstadt, Germany). ^35^ S incorporation was measured in a scintillation counter and calculated per DNA amount, which was determined in samples with Quant-iT™ PicoGreen® dsDNA Reagent (Molecular Probes, Inc.) according to the manufacturer’s protocol.

### Measurement of tyrosine kinase activity of EGF receptor

Cells were seed in a number of 10^4^ cells per well of 96-well plate. Following an overnight incubation, standard growth medium was substituted with the medium supplemented with appropriate concentrations of genistein synthetic derivatives or a potent tyrosine kinase inhibitor PD168390 (Merck KGaA, Darmstadt, Germany), and cells were incubated for 2 hours. Then, Epidermal Growth Factor (BD Biosciences, Franklin Lakes, NJ USA) was added to 100 ng/ml to induce EGF receptor autophosphorylation. After 15 min the medium was removed, and cells were fixed with 4% formaldehyde. Tyrosine kinase activity of EGF receptor was assessed using commercially available Cell-Based ELISA, Human Phospho-EGFR (Y1068) Immunoassay (R&D Systems, Inc) according to the manufacturer’s protocol.

### Electron microscopic studies

Cells were incubated in growth medium supplemented with appropriate concentrations of tested compounds for 6 days. Following PBS washing, cells were fixed with 2.5% glutaraldehyde, and then with 1% osmium tetroxide and 1% potassium hexacyanoferrate (III) followed by ethanol dehydration. Sections of Epon 812 resin (Fluka, Germany) embedded cells were stained in lead citrate and uranyl acetate and examined under transmission electron microscope (Philips CM100). The number of different lysosomal structures was determined.

### Computational prediction of BBB penetration

Following physicochemical parameters were determined for each synthetic derivative of genistein: molecular weight (MW), octanol/water partition coefficient (cLogP), topological polar surface area (tPSA), the number of hydrogen bond donors (HBD) and hydrogen bond acceptors (HBA). Calculation of cLogP was performed on the basis of the chemical structure of compounds using ALOGPS 2.1 Program (VCCLAB) accessible via Internet (http://www.vcclab.org) and assessment of tPSA was performed with MarvinSketch 5.2.6 (ChemAxon Ltd.) accessible via Internet (http://intro.bio.umb.edu/111-112/OLLM/111F98/newclogp.html). Predicted logBB was assessed as proposed previously [28].

### Statistical analysis

Effects of different concentrations of genistein synthetic derivatives on the number of lysosomal structures was tested by one-way ANOVA with Tukey’s multiple comparisions as a post-hoc test. Statistical tests were performed using Statistica 8.0 [StatSoft, Poland] software with significance at *p* < 0.05.

## Results

### Cytotoxicity and antiproliferative activity of genistein synthetic derivatives

We have tested 32 synthetic derivatives of genistein in order to establish cytotoxicity and antiproliferative activity in cultured fibroblasts after 24 h exposition to these compounds in a concentration range 1–30 μM. We found toxicity of several synthetic derivatives as low as that of genistein (compounds: IFG-032, IFG-034, IFG-036, IFG-038, IFG-053, IFG-054, IFG-066, IFG-070, IFG-071, IFG-072), while others exhibited higher cytotoxicity (see LC_50_ values, Table 2). Additionally, most of the compounds with low cytotoxicity (excluding IFG-053 and IFG-070) exhibited antiproliferative activity similar to or lower than that of genistein (see IC_50_ values, Table 2).

**Table 2:** Cytotoxic and antiproliferative activities of genistein synthetic derivatives and their effects on kinetics of GAG synthesis in fibroblasts

**Compound**	**Cytotoxicity LC**_**50**_**[μM]**	**Proliferation IC**_**50**_**[μM]**	**GAG synthesis [%]**
IFG-001	13.9	-	120 ± 56
IFG-018	41.7	-	76 ± 38
IFG-021	11.0	-	75 ± 25
IFG-027	13.1	-	69 ± 46
IFG-032	n/c	17.4	57 ± 24
IFG-034	n/c	22.9	65 ± 19
IFG-035	22.6	-	93 ± 70
IFG-036	n/c	11.2	64 ± 21
IFG-037	23.5	-	88 ± 84
IFG-038	n/c	19.9	55 ± 13
IFG-042	22.0	-	48 ± 29
IFG-043	14.5	-	46 ± 41
IFG-046	20.7	-	118 ± 72
IFG-048	20.3	-	73 ± 5
IFG-050	11.6	-	100 ± 65
IFG-051	14.6	-	127 ± 109
IFG-052	15.8	-	74 ± 19
IFG-053	n/c	53.0	92 ± 52
IFG-054	n/c	n/a	119 ± 64
IFG-060	14.9	-	31 ± 8
IFG-061	18.8	-	50 ± 25
IFG-062	12.1	-	403 ± 206
IFG-063	51.5	-	76 ± 6
IFG-064	6.6	-	133 ± 77
IFG-065	22.7	-	96 ± 22
IFG-066	n/c	14.6	34 ± 10
IFG-067	15.1	-	96 ± 38
IFG-070	n/c	48.1	101 ± 30
IFG-071	n/c	17.9	49 ± 20
IFG-072	n/c	14.1	79 ± 42
IFG-073	27.5	-	83 ± 32
IFG-074	44.1	-	88 ± 39
Genistein	n/c	16.9	55 ± 31

Cytotoxicity is expressed as LC_50_ index value, i.e. concentration of the tested drug [μM] that is lethal to 50% of cells in a culture exposed to the tested compound for 24 hours. Antiproliferative activity is expressed as IC_50_ index value, i.e. concentration of the tested drug [μM] that causes 50% inhibition of cell proliferation in a culture exposed to the tested compound for 7 days. Kinetics of GAG synthesis is expressed as relative ^35^ S incorporation into GAGs after 3-day exposure to derivatives of genistein at 30 μM concentration. Labeling was conducted for 24 hours with 20 μCi/ml H_2_[^35^ S]O_4_. Radioactivity of incorporated ^35^ S was measured in a scintillation counter, calculated per DNA amount [dpm/ng DNA] and expressed as the percentage of control, where 100% corresponds to the relative ^35^ S incorporation into control (cell culture treated with 0.05% dimethylformamide).

**n/c** denotes experiments in which no cytotoxicity was observed (over 90% of cells survived at concentrations ranging from 1 to 30 μM).

**n/a** denotes experiments in which no antiproliferative activity was observed (relative proliferation was over 95% at concentrations ranging from 1 to 30 μM).

**-** denotes experiments in which IC_50_ for antiproliferative activities could not be determined due to cytotoxic effects of the tested compounds.

### Reduction of GAG synthesis by some genistein synthetic derivatives

Since genistein inhibits GAG synthesis in a dose-dependent manner, we have tested the effects of 32 genistein synthetic derivatives on GAG synthesis in fibroblast cultures. The level of GAG synthesis was estimated by measurement of [^35^ S]O_4_^2-^ uptake. Different genistein synthetic derivatives revealed various ability to inhibit GAG synthesis, though six of them (IFG-032, IFG-038, IFG-042, IFG-043, IFG-061, IFG-071) decreased GAG synthesis after 3-day exposure to extent similar to that of genistein, while two (IFG-060, IFG-066) were even more effective (Table 2). Interestingly, some compounds (IFG-001, IFG-046, IFG-051, IFG-054, IFG-062, IFG-064) stimulated rather than inhibited GAG synthesis, with IFG-062 being a strong (over 4-fold) stimulator (Table 2).

### Decrease of lysosomal storage of GAGs by some genistein synthetic derivatives

**Figure 1:**
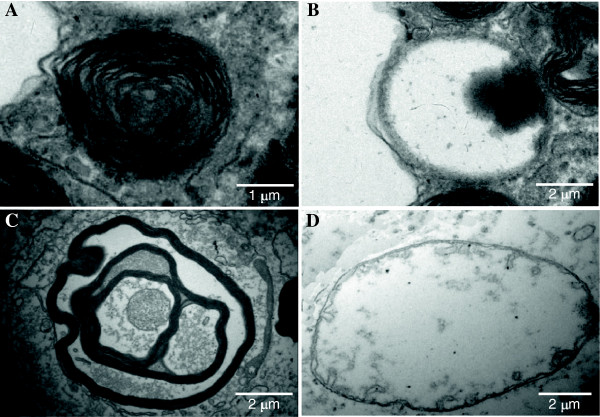
Electron microphotographs of different types of lysosomal structures in MPS III fibroblasts: lysosome with lamellar and electron-dense structure (A), lysosome of amorphous, flocculent and electron-lucent structure (B), complexed lysosomal structure (autophagolysosome) with storage material of different electron density (C), and vacuoles of unknown origin and function (D).

For further studies, we have selected the derivatives which revealed: (i) low cytotoxicity, (ii) antiproliferative activity similar to genistein, and (iii) efficient inhibition of GAG synthesis. Assuming that reduction in GAG synthesis may lead to a decrease in lysosomal storage, as concluded previously [13,14], we assessed the storage in MPS cells by using electron microscopic techniques. The number of different lysosomal structures were counted and calculated per 100 μm^2^ of cell cross-section. Examples of abnormal storage structures, observed in MPS IIIA and MPS IIIB cells, are presented in Figure 1.

We have observed a statistically significant decrease in the number of different abnormal lysosomal structures in MPS IIIA or MPS IIIB fibroblasts after 6-day exposure to all selected genistein synthetic derivatives at 30 μM, relative to untreated cells (Table 3). Average size of these structures did not change significantly (data not shown). Interestingly, unusual large vacuole-like structures of unknown function or content, were observed in cells treated with compound IFG-066 (Figure 1D).

**Table 3:** Effect of synthetic derivatives of genistein at 30 μM concentration on the number of different lysosomal structures in MPS IIIA and MPS IIIB fibroblasts

**Compound**	**Number of structures per 100 μm**^**2**^**of cellular cross section ± SD**			
	**lamellar**	**complexed**	**amorphous**	**total number**
	**MPS IIIA**			
None	0.40 ± 0.17	0.35 ± 0.35	0.38 ± 0.27	1.13 ± 0.50
IFG-032	0.14 ± 0.08 *	0.22 ± 0.11	0.17 ± 0.16 *	0.53 ± 0.19 *
IFG-034	0.17 ± 0.09 *	0.27 ± 0.14	0.17 ± 0.09 *	0.61 ± 0.20 *
IFG-036	0.19 ± 0.14 *	0.28 ± 0.16	0.22 ± 0.11	0.73 ± 0.31 *
IFG-038	0.32 ± 0.16	0.27 ± 0.13	0.21 ± 0.13	0.80 ± 0.24 *
IFG-066	0.13 ± 0.10 *	0.16 ± 0.08	0.18 ± 0.19 *	0.48 ± 0.17 *
IFG-071	0.28 ± 0.13	0.26 ± 0.09	0.25 ± 0.11	0.79 ± 0.22 *
IFG-072	0.20 ± 0.10 *	0.28 ± 0.10	0.34 ± 0.17	0.82 ± 0.23
	**MPS IIIB**			
None	0.44 ± 0.18	0.14 ± 0.10	0.17 ± 0.12	0.75 ± 0.31
IFG-032	0.17 ± 0.11 *	0.10 ± 0.07	0.12 ± 0.08	0.40 ± 0.20 *
IFG-034	0.20 ± 0.16 *	0.06 ± 0.05	0.15 ± 0.08	0.41 ± 0.16 *
IFG-036	0.25 ± 0.21 *	0.12 ± 0.11	0.15 ± 0.18	0.50 ± 0.34
IFG-038	0.21 ± 0.12 *	0.10 ± 0.10	0.09 ± 0.09	0.41 ± 0.18 *
IFG-066	0.15 ± 0.10 *	0.11 ± 0.14	0.08 ± 0.06	0.34 ± 0.23 *
IFG-071	0.07 ± 0.07 *	0.07 ± 0.06	0.13 ± 0.09	0.26 ± 0.13 *
IFG-072	0.11 ± 0.12 *	0.16 ± 0.08	0.14 ± 0.10	0.41 ± 0.20 *

Asterisks (*) indicate statistically significant differences (one-way ANOVA with Tukey’s multiple comparisions as a post-hoc test, p < 0.05) relative to control MPS IIIA and MPS IIIB cells (None) where no tested compound was added into culture medium.

### Phosphorylation of EGF receptor in the presence of genistein synthetic derivatives

**Figure 2:**
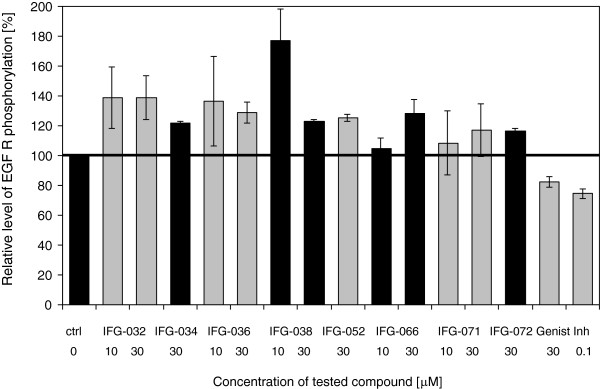
**Effect of selected synthetic derivatives of genistein on the activity of tyrosine kinase of EGF R.** Relative levels of EGF R phosphorylation relative to untreated control cells (ctrl), genistein (Genist) and artificial inhibitor - PD168390 (Inh), with bars indicating standard deviation between two independent experiments, is presented. Statistically significant (p < 0.05) decrease in relative level of EGFR phosphorylation, relative to the control cells (ctrl) was detected only for genistein (at 30 μM) and PD168390 (at 0.1 μM).

Reduction of GAG synthesis is assumed to be the result of impaired expression of genes coding for enzymes required for GAG production, and involves an intracellular signaling pathway depending of tyrosine kinase activity of EGFR [13,29]. Since genistein was reported to inhibit the activity of tyrosine kinase of EGFR, and thus to impair production of GAGs, we have tested the ability of some genistein synthetic derivatives (able to inhibit GAG synthesis) to impair phsophorylation of EGFR. We have observed that none of the tested synthetic derivatives of genistein affects the tyrosine kinase activity of EGF R. Comparing to genistein or a potent tyrosine kinase inhibitor PD168390, no decrease of phosphorylation of EGFR was observed (Figure 2), suggesting that the synthesis of GAGs is reduced by investigated compounds by some other, unknown, mechanism(s).

### Computational prediction of BBB penetration

Because genistein was reported to cross BBB only to some extent (several percent efficiency) we aimed to develop new derivatives of genistein with more lipophilic properties that could be able to cross this barrier more readily. Chemical modifications of the genistein molecule resulted, in most of the cases, in an increase of cLogP values, suggesting an increase in lipophilicity (Table 4). Moreover, some of genistein derivatives revealed tPSA values optimal for compounds able to cross BBB (tPSA < 90 Å^2^) (Table 4). These results suggest a possible improvement in physicochemical properties of some modified genistein molecules compared to unmodified ones, in the light of efficiency of crossing BBB.

**Table 4:** **Physicochemical properties of synthetic derivatives of genistein assessed*in silico***

**Compound**	***MW***	**cLogP**	**tPSA**	**HBA**	**HBD**	**LogBB**
Genistein	270.24	2.27	89.82	5	3	−0.86
IFG-001	389.37	4.23	121.91	7	4	−1.12
IFG-018	508.65	8.64	93.06	6	2	0.08
IFG-021	770.69	3.23	235.18	18	2	−2.97
IFG-027	310.30	3.45	75.99	5	2	−0.46
IFG-032	396.35	2.10	105.20	8	0	−1.10
IFG-034	432.39	4.20	119.36	8	2	−0.98
IFG-035	390.43	5.55	53.99	5	0	0.19
IFG-036	356.33	3.04	102.29	7	2	−0.91
IFG-037	384.38	3.85	102.29	7	2	−0.79
IFG-038	625.10	4.11	133.19	8	3	−1.20
IFG-042	498.52	5.44	117.59	9	1	−0.77
IFG-043	360.36	4.40	75.99	5	2	−0.31
IFG-046	556.14	3.74	146.20	10	2	−1.45
IFG-048	384.38	3.85	105.12	7	2	−0.83
IFG-050	326.30	2.64	88.52	6	2	−0.77
IFG-051	400.42	5.58	64.99	5	1	0.03
IFG-052	472.44	5.38	108.36	8	1	−0.64
IFG-053	584.66	8.28	112.57	7	1	−0.26
IFG-054	328.27	2.05	116.12	7	3	−1.27
IFG-060	474.47	4.51	122.19	8	2	−0.98
IFG-061	496.44	3.37	108.36	7	1	−0.95
IFG-062	390.39	4.46	85.22	6	2	−0.44
IFG-063	354.31	3.30	102.29	7	2	−0.87
IFG-064	402.44	5.67	64.99	5	1	0.04
IFG-065	444.52	6.69	53.99	5	0	0.36
IFG-066	312.32	3.54	78.82	5	2	−0.49
IFG-067	402.40	4.43	82.06	6	1	−0.40
IFG-070	342.16	2.67	102.29	7	2	−0.97
IFG-071	398.40	3.92	102.29	7	2	−0.78
IFG-072	328.31	2.44	96.22	6	3	−0.91
IFG-073	314.22	2.17	96.22	6	3	−0.95
IFG-074	569.73	4.17	116.12	7	3	−0.94

**MW** – molecular weight [Da], **cLogP** – calculated octanol/water partition coefficient, **tPSA** – topological polar surface area [Å^2^], **HBA** – hydrogen bond acceptor number (O + N), **HBD** – hydrogen bond donor number (OH + NH), **LogBB** – predicted BBB permeation.

## Discussion

An important issue in development of therapeutic approaches for MPS types with neurological symptoms is the ability of potential therapeutic agents to cross BBB. Enzyme replacement therapy, a treatment based on systematic, intravenous administration of the lacking enzyme, although effective - to some extent - in treatment of visceral organs, is of low efficacy in treatment of cases where the central nervous system is affected [1,6]. Alternative therapeutic approaches, such as substrate reduction therapies, are based on assumptions that low-molecular-weight molecules might be able to cross BBB and penetrate the brain readily [29].

The results presented in this report indicate that some synthetic derivatives of genistein, particularly, IFG-060 and IFG-066, are potent inhibitors of GAG synthesis. Impairment of GAG synthesis by IFG-032, IFG-034, IFG-036, IFG-038, IFG-066, IFG-071 and IFG-072 was also an effective method for reduction of lysosomal storage in MPS IIIA and/or MPS IIIB cell cultures, as it was previously reported for genistein [7]. Studies on MPS IIIB mice suggested that GET IT may be a promising treatment [16,17]. Thus, according to results obtained in this study, we suggest that artificial genistein derivatives listed in Table 3 might be considered as potential drugs to be used in treatment of MPS.

In the development of new therapies, it is crucial for a potential drug to be safe for humans. In this study, some synthetic derivatives of genistein (including the efficient reducers of GAG storage, listed in Table 3) revealed low cytotoxicity and minor effects on cell proliferation. This appears important in the light of safety problems with another effective inhibitor of GAG synthesis, rhodamine B [30,31]. Therefore, it seems that some derivatives of genistein (e.g. IFG-032, IFG-034, IFG-036, IFG-038, IFG-066, IFG-071, IFG-072) possess desirable biological properties for a potentially safe and effective drug. Moreover, predicted changes of physicochemical properties of some synthetic derivatives, relative to genistein (as assessed *in silico*), might result in improvement of ability to cross BBB (see Table 4). On the other hand, it is necessary to stress that such an improved crossing of BBB was only calculated *in silico* by using algorithms based on putative physicochemical properties of compounds, predicted from their formulas, according to previously described models [28]. One has to consider that such an approach, although based on solid physical and chemical assumptions, cannot reflect all biological processes, among which a possible active transport of tested compounds may be especially important. Therefore, it should be noted that for determination of actual abilities of penetration of BBB by all compounds described in this report, it will be necessary to perform experiments with either BBB models or (preferably) laboratory animals. Synthesis of labeled isoflavones should be the first step in the way to assess the real (not only calculated or predicted) efficiency of BBB penetration by tested genistein derivatives.

Interestingly, the mechanism of action by which selected synthetic derivatives of genistein inhibit GAG production seems to be different from that described previously for genistein. Namely, contrary to this natural isoflavone, its artificial derivatives did not affect the EGF-dependent pathway, as they were not able to inhibit the EGFR kinase activity. It is worth noting that similar phenomenon was observed for various natural flavonoids causing GAG synthesis inhibition [24]. It is, therefore, tempting to speculate that various chemical modifications of the genistein molecule destroy its activity of the EGFR kinase inhibitor, while either retaining/enhancing or gaining a new function of GAG synthesis inhibitor by influencing another, as yet unidentified, biochemical pathway.

Finally, one should note that apart from genistein derivatives that decreased GAG synthesis, there were also compounds significantly enhancing the efficiency of this process, like IFG-062. Therefore, we assume that the set of artificial genistein derivatives described in this report might be a useful tool in further studies on molecular mechanisms of regulation of GAG synthesis.

## Conclusions

Some synthetic derivaties of genistein revealed low cytotoxicity and small (if any) effects on cell proliferation, while slowing down GAG synthesis (though by a pathway other than inhibition of EGF receptor’s tyrosine kinase activity) and decreasing lysosomal storage. These compounds had higher potential abilities to cross BBB than genistein. Thus, we suggest they are promising candidates for further studies on therapeutic agents in MPS types with neurological symptoms.

## Abbreviations

BBB, Blood–brain barrier; EGF, Epidermal growth factor; EGFR, Epidermal growth factor receptor; ERT, Enzyme replacement therapy; GAG(s), Glycosaminoglycan(s); GET IT, Gene expression-targeted isoflavone therapy; MPS, Mucopolysacccharidoses; SRT, Substrate reduction therapy.

## Competing interest

Genistein and its derivatives listed in Table 1 have been claimed as isoflavones for treating MPS in the United States Patent no. US 8,178,609 B2 (date of patent: May 15, 2012; inventors: Grynkiewicz G., Wegrzyn G., Szechner B., Tylki-Szymanska A., Wegrzyn A., Jakobkiewicz-Banecka J., Baranska S., Czartoryska B., Piotrowska E.; title: Isoflavones for treating mucopolysaccharidoses). The authors declare no other competing interest.

## Authors’ contributions

AK performed all experiments but electron microscopic studies, and performed both *in silico* and statistical analyses; MN designed electron microscopic experiments, executed them, analyzed their results and interpreted them; JJB designed other experiments, interpreted their results and participated in drafting the manuscript; GG and WS designed and performed syntheses of genistein derivatives; MGC analyzed the results and participated in drafting the manuscript; GW planned the study, coordinated the project, drafted the first version of the manuscript and prepared its final version. All authors read and approved the final manuscript.
